# Deterioration Process of Concrete Exposed to Internal Sulfate Attack

**DOI:** 10.3390/ma13061336

**Published:** 2020-03-15

**Authors:** Weifeng Chen, Bei Huang, Yuexue Yuan, Min Deng

**Affiliations:** 1 College of Mateials Science and Engineering, Nanjing Tech University, Nanjing 211800, China; chenweifeng@njtech.edu.cn (W.C.); yxynjtech@126.com (Y.Y.); dengmin@njtech.edu.cn (M.D.); 2 State Key Laboratory of Materials-Oriented Chemical Engineering, Nanjing 211800, China

**Keywords:** internal sulfate attack, concrete, gypsum, performance

## Abstract

Damage to concrete structures with gypsum-contaminated aggregate occurs frequently. Aggregates in much of the southern part of China are contaminated with gypsum. Therefore, in this study, the effects of using different quantities of gypsum-contaminated aggregate on the expansion and compressive strength of concrete were investigated over a period of one year. Two groups of concrete were designed with the gypsum-contaminated aggregate containing different parts of fine and coarse aggregate, respectively. The SO_3_ contents were 0%, 0.5%, 1%, 1.5%, 3%, 5%, and 7% by weight of aggregate. X-ray diffraction (XRD), thermogravimetry (TG), and differential scanning calorimetry (DSC) were used to analyze the change in mineral composition over time. The microstructure was also studied by scanning electron microscopy (SEM) and energy dispersive spectrometry (EDS). The results showed that significant expansion and great loss in compressive strength did not occur in concrete if the content of SO_3_ lay below 1.5% and 3% in fine and coarse aggregates, respectively. The concentration of sulfate ions in concrete was not enough to form new a phase of gypsum. During the process of internal sulfate attack, the content of gypsum decreased and the content of ettringite increased. Ettringite was the main reason for the expansion damage of concrete. Additionally, the fracture mode of internal sulfate attack on concrete was the crack extension from gypsum to paste; finally, the aggregate separated from the paste.

## 1. Introduction

Sulfate attack on concrete is a key durability problem. Internal sulfate attack (ISA) on concrete is a reaction between sulfates present in the original mix of concrete and the calcium aluminate in cement and water, which can show signs of deterioration [1]. In the early stage of hydration, the gypsum reacts with tricalcium aluminate to form ettringite (AFt), which does not damage concrete. It is beneficial and leads to an increase in strength. However, the excess gypsum remains in the hardened concrete, and the gypsum will dissolve sulfate ions. The SO_4_^2−^ continues to react with the remaining tricalcium aluminate to form AFt, which is harmful to concrete [2]. The harmful AFt may cause a deleterious expansion and strength loss, since large stresses are generated in the hardened cement paste [3]. In the past 30 years, ISA damage due to aggregates with sulfate salts has been found in tunnels and dams in China [4], Middle Eastern countries [5], and some European countries [6]. A large number of engineering disasters have occurred because of the aggregate contained in sulfates. As a result, great economic losses as well as safety problems have arisen.

The mechanism of external sulfate attack (ESA) has been studied for many years. Sulfate is supplied by seawater, groundwater, and industrial wastewater. Sulfate ions react with ionic species of the pore solution to precipitate ettringite, gypsum, or thaumasite [7]. The major reaction product of ESA is AFt. The formation of AFt results in expansion and cracking, spalling, and other damaging effects. There is a correlation between the C_3_A content in Portland cement and the susceptibility of concrete to sulfate attack, and cements with limited C_3_A content were introduced to the market. These cements are used in the concrete that is exposed to moderate and severe sulfate concentrations [8,9]. The sulfate ions react with portlandite to form gypsum. Even though gypsum formation is generally accepted to be harmful, the specific mechanism by which this occurs is not well established. It is widely accepted that gypsum formation only has a softening effect and causes mass and strength loss. However, Tian demonstrated that gypsum formation during sulfate attack may cause expansion [10]. The development of gypsum is influenced by the pH value and the concentration of sulfate ions. In sulfate solution, the minimum concentration of sulfate ions to form gypsum is 1400 mg/L, at a pH value equal to 12.4 [11]. Normally, gypsum is the first reaction product at a high sulfate ion concentration, especially SO_4_^2−^ > 8000 mg/L [12]. Thaumasite is usually considered to be formed at a low temperature in the presence of carbonate [13]. The thaumasite form of sulfate attack is different from ettringite formation because it is the C-S-H in hardened Portland cement that is targeted for the reaction and not the calcium aluminate hydrates. However, C-S-H is the primary binding agent in concrete [14].

Compared with the sulfate ions that penetrate into concrete by diffusion for ESA, there is a higher concentration of sulfate ions in concrete for ISA. The hazard is illustrated in the materials which cause higher tensile stress, leading to spalling and disruption of concrete structures [15]. For example, several highway tunnels in China were damaged seriously by ISA in the year after they were built. In order to prevent ISA, Chinese national standards have set the upper limit for the SO_3_ content in fine and coarse aggregates. It is stipulated in GB/T14684 and GB/T14685 that the SO_3_ content in fine and coarse aggregates shall not exceed 0.5% and 1%, respectively. Samarai proposed a 0.1% expansion as a safe margin for finding the allowable sulfate level [16]. According to Crammond, the allowable gypsum content in aggregates used in Portland cement mortars would not be much larger than 2.5% by weight of aggregate [17]. Because of the higher concentration of sulfate ions, the types of reaction products and the degradation process of ISA are important problems to be studied. In this paper, the expansion, strength loss, and formation process of reaction products of concrete with different amounts and particle sizes of gypsum-contaminated aggregate were investigated, and we aimed to discuss the feasibility of using gypseous rock as a concrete aggregate for highways in Shanxi province.

## 2. Research Significance

In this paper, the influences of the dosage of gypsum aggregate on the compressive strength and expansion performance of concrete were studied. The types of reaction products in the process of internal sulfate attack were also studied.

## 3. Materials and Mixes

### 3.1. Raw Materials

**Figure 1:**
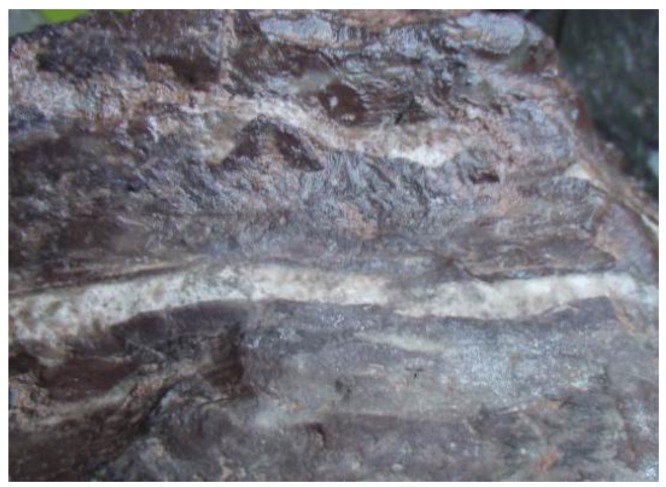
Appearance of gypsum-contaminated aggregate.

**Figure 2:**
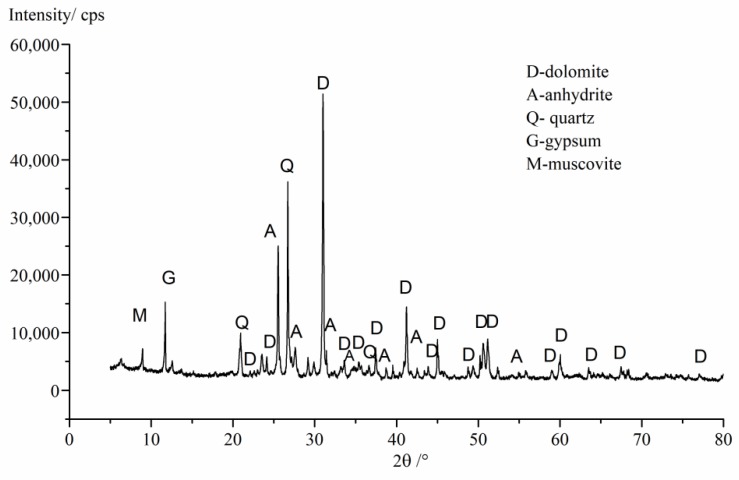
XRD patterns of gypsum-contaminated aggregates.

P·II 52.5 Portland cement conforming to Chinese National Standard GB175 was used in this study and its chemical composition is shown in Table 1. Two types of aggregate were used. One was gypseous aggregate, which was crushed into fine and coarse aggregate, from the WU YAN HE railway tunnel from Shanxi province to Shandong province. Another was normal aggregate with no gypsum; river sand was used as fine aggregate and limestone was used as coarse aggregate. The appearance of gypsum-contaminated aggregate is shown in Figure 1. The chemical compositions and XRD patterns of this aggregate are shown in Table 2 and Figure 2, respectively. The chemical compositions of white part (WP) are shown in Table 3. From analysis results, it was found that the WP of this rock was a mix of gypsum and anhydrite, and the rock matrix was dolomite.

### 3.2. Mix Proportion

In our experimental program, there were two types of concrete specimen: 100 mm by 100 mm by 100 mm and 75 mm by 75 mm by 280 mm. They were demolded a day after casting and cured in water at 20 ± 2 °C. The cube samples were used to determine the compressive strength of the concrete with different amounts and sizes of gypsum. The prism specimens were used for the ultrasonic and expansion test of concrete at different ages. 

**Figure 3:**
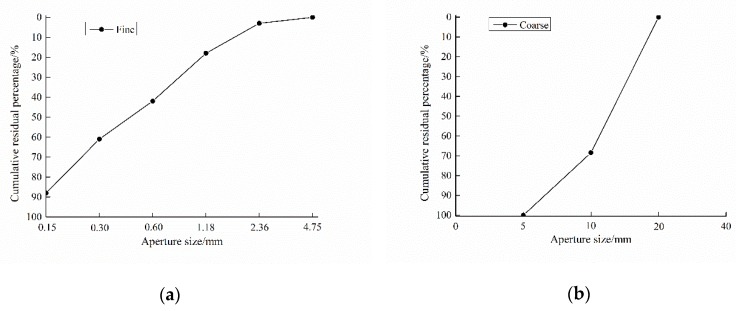
The grain size curve of aggregates: (**a**) fine; (**b**) coarse.

The gypsum-contaminated aggregate was crushed into gypsum sand (<4.75 mm) and gypsum stone (5–20 mm). Part of the normal fine and coarse aggregate was replaced with gypsum-contaminated aggregate in the mixture of concrete, to get five levels of SO_3_ content (0, 0.5%, 1.0%, 1.5%, 3.0%, 5.0%, and 7% by weight of aggregate) in the concrete. The grain size curves of fine and coarse aggregates are shown in Figure 3. The mix design of concretes is shown in Table 4.

## 4. Methods

### 4.1. Expansion Test on Concrete

**Figure 4:**
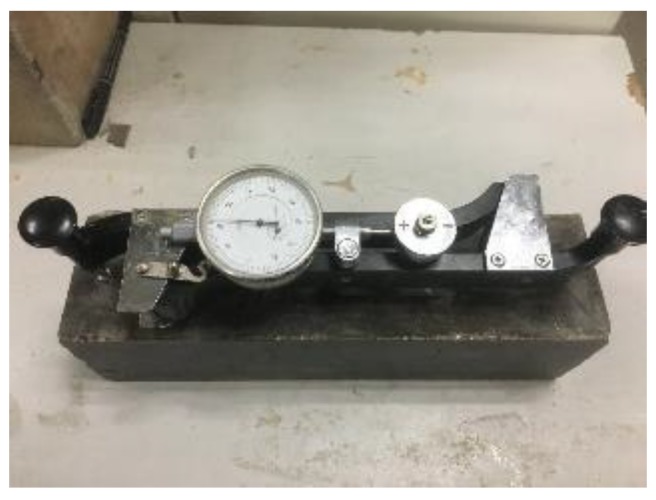
Expansion test on concrete.

An expansion test was conducted using three specimens per mix. The reference points used to measure the dimensional variations of samples were placed on the 75 mm by 280 mm sides that were in contact with the lateral sides of the molds. As shown in Figure 4, two reference points were placed on each side with 250 mm separation. According to Equation (1), the expansion of a specimen at a certain age was calculated by the average of the measurements performed at both sides with a handholding strain gauge with 0.01% precision. In the formula, *L*_0_, *L*, and *L_t_* represent the initial length, effective length of 250 mm, and testing length at different ages, respectively.

(1) $$P=\frac{L_{t}-L_{0}}{L}\times 100\%$$

### 4.2. Compressive Strength of Concrete

**Figure 5:**
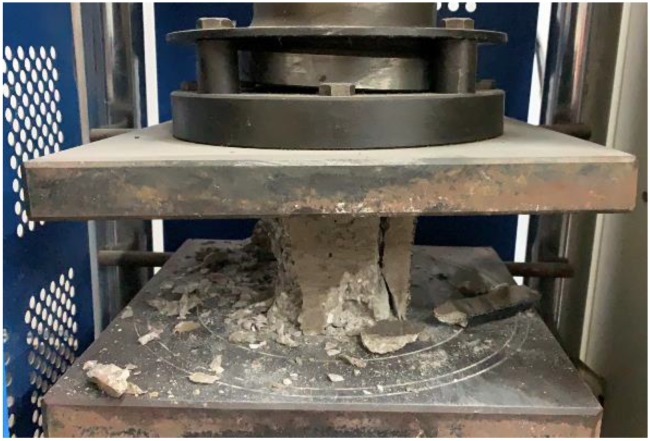
Compressive strength test on cubic concrete.

The compressive strength of the concrete cubic specimen was examined at the ages of 28, 60, 90, 150, 210, and 240 days by using a compressive-testing machine with a loading rate 0.5–0.8 MPa/s. The machine for testing compressive strength is shown in Figure 5. For each test, the average compressive strength of the three cubic concrete samples was used. 

### 4.3. Ultrasonic Test on Prism Concrete

**Figure 6:**
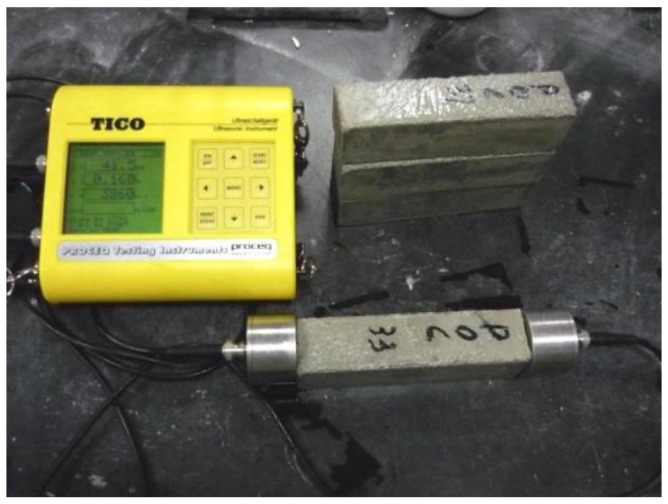
Ultrasonic pulse velocity test on prism concrete.

The ultrasonic velocities, which describe the material internal damage, in specimens at different ages were tested, by fixing the launcher and receiver transducer firmly on the two sides of the specimen (75 mm by 75 mm), in accordance with the “Technical specification for inspection of concrete defects by ultrasonic method” (CECS 21: 2000). The machine used to test the ultrasonic pulse velocity is shown in Figure 6.

### 4.4. XRD and Thermogravimetry–Differential Scanning Calorimetry (TG–DSC) Analysis on Mortar

To get the complementary mineral information, mortar specimens were subjected to XRD and TG–DSC analyses at different ages. The mortars cut from the concrete samples were immersed in absolute ethyl alcohol for 12 h to terminate hydration, before being vacuum dried at 60 °C for 24 h, and then ground into powder. The test powders were passed through an 0.08 mm sieve. The XRD data were collected in the range of 10°–70°, 2θ at a counting time of 15 s/step, and a divergence slit of 1°. TG–DSC data were obtained from 50 to 950 °C at a rate of temperature increase of 10 °C/min in a N_2_ atmosphere.

### 4.5. Microstructure Examination on Concrete

In order to investigate the morphology of concrete under internal sulfate attack, a fractured fresh piece of concrete was sampled, hydration terminated, and vacuum dried. Then, the dried sample was embedded in a low-modulus epoxy, polished using progressively smaller grids, coated with a gold-palladium coating, and investigated using JSM-7600F SEM.

### 4.6. Dissolution of WP in Concrete

The pore solution in concrete was expressed directly with a compressive-testing machine whose final pressure value was 2000 MPa at a speed of 2 MPa/s. Then, the sulfate concentration in this pore solution was tested by inductive coupled plasma emission spectrometer (ICP). 

## 5. Results

### 5.1. Expansion Measurements

**Figure 7:**
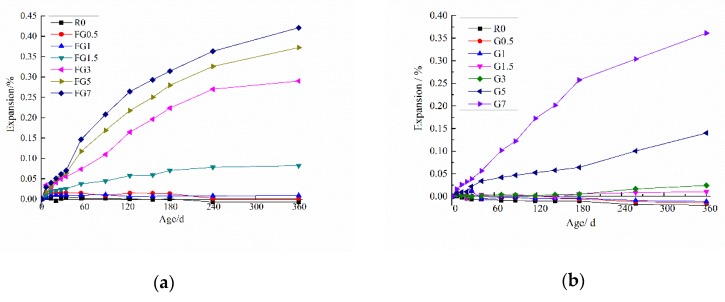
Expansion curves of concrete with gypsum-contaminated aggregate: (**a**) gypsum-contaminated fine aggregate; (**b**) gypsum-contaminated coarse aggregate.

Figure 7a presents the expansion data for the concretes with different amounts of gypsum-contaminated fine aggregate. Figure 7b shows the change in length of concrete with different amounts of contaminated coarse aggregate. The results for the gypsum-contaminated fine aggregate samples show that the mixes with a gypsum content above 3% SO_3_ presented a significant increase in expansion for 360 days. There was a little expansion when the SO_3_ content in sand was 1.5%, and the expansions of the specimen was less than 0.1% in 360 days. Meanwhile, specimens containing SO_3_ less than 1.5% showed no expansion. 

For the samples with gypsum-contaminated coarse aggregate, no significant variation was observed, when the SO_3_ content in coarse aggregate was less than 3%. The average expansion increased obviously as the SO_3_ content increased to 5% and 7%. The final measured expansions for the specimens containing 5% and 7% SO_3_ were 0.1401% and 0.3602%, respectively, at 360 days. The expansion data in Figure 7a indicated that the smaller aggregate particle size resulted in higher expansion of the specimen, at the same SO_3_ content.

Several studies recommended 0.1% expansion as a safe margin for determining the maximum percentage of sulphate than can be present in a mixture without causing any significant degradation [2]. Based on the results shown in Figure 7, FG1.5, FG1, FG0.5, G3, G1.5, G1, and G0.5 comply with the maximum expansion suggested in the literature. Consequently, these mixtures should not show a risk of damage from a structural point of view, but these SO_3_ contents are bigger than the limit established in several standards, such as GB/T14684 an GB/T14685 in China.

### 5.2. Compressive Strength

**Figure 8:**
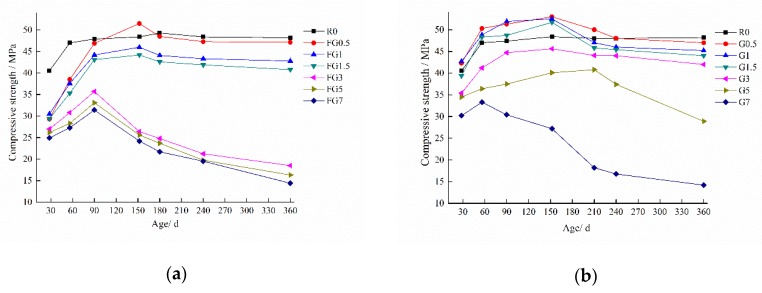
Compressive strength of concrete with gypsum-contaminated aggregate: (**a**) gypsum-contaminated fine aggregate; (**b**) gypsum-contaminated coarse aggregate.

Figure 8 shows the compressive strength development for concrete with various amounts of gypsum-contaminated aggregate. The results indicate that all concrete specimens exhibited a reduction in initial compressive strength as SO_3_ increased. It was evident that with 0.5–1.5% SO_3_ in sand, the initial compressive strength of concrete decreased by 25%. Additionally, 3–7% SO_3_ in sand led to a loss in the initial compressive strength of samples of about 38%. The compressive strength of all specimens with gypsum-contaminated aggregate increased first and then decreased with the curing age. As shown in Figure 8a, the final strengths of the FG0.5, FG1.0, and FG1.5 specimens at 360 days were higher than the initial strength of the reference sample, which means that an SO_3_ content in sand of less than 1.5% is relatively safe. 

For the samples with SO_3_ in coarse aggregate, only the strength of the G7 specimen increased—from 30.2 to 33.3 MPa in 28 days—before reducing to 14.2 MPa at 360 days because of the expansion properties shown in Figure 7. The strength curve of the G5 sample showed a significant decrease after 210 days. It was indicated that samples with gypsum incorporated (0.5–3% SO_3_) had less effect on the compressive strength of concrete relative to the initial strength of the reference.

### 5.3. Ultrasonic Test

**Figure 9:**
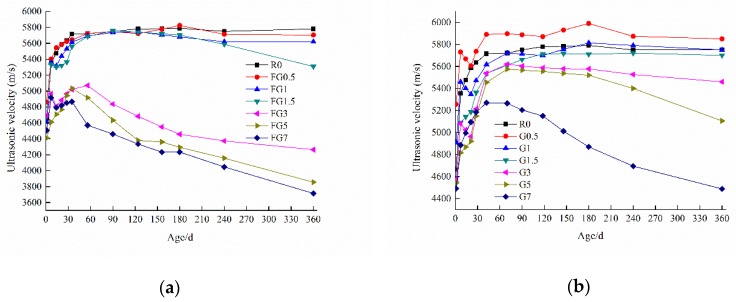
Ultrasonic test results of concrete samples: (**a**) gypsum-contaminated fine aggregate; (**b**) gypsum-contaminated coarse aggregate.

The internal damage of specimens under internal sulfate attack was tested by ultrasonic pulse velocity, and the results are presented in Figure 9. The value of ultrasonic velocity is closely related to the microstructure. The more compact a structure is, the higher the ultrasonic velocity obtained will be. The results show that the change trend for the ultrasonic velocities of samples is consistent with that of compressive strength. Except for the FG3, FG5, FG7, G5, and G7 specimens, the damage to the other samples was small.

### 5.4. Mineral Composition Examination

**Figure 10:**
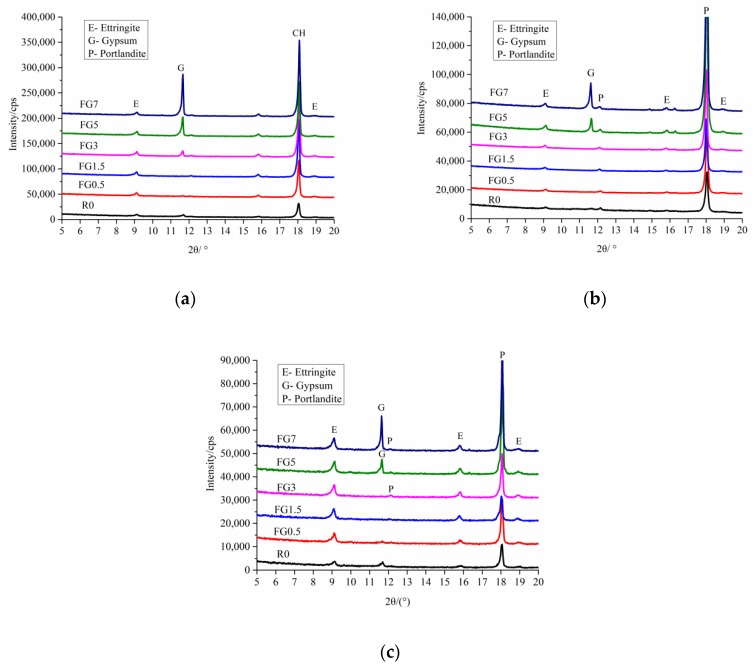
XRD patterns of paste in concrete with gypsum-contaminated fine aggregate of different ages: (**a**) 56 days; (**b**) 240 days; and (**c**) 360 days.

**Figure 11:**
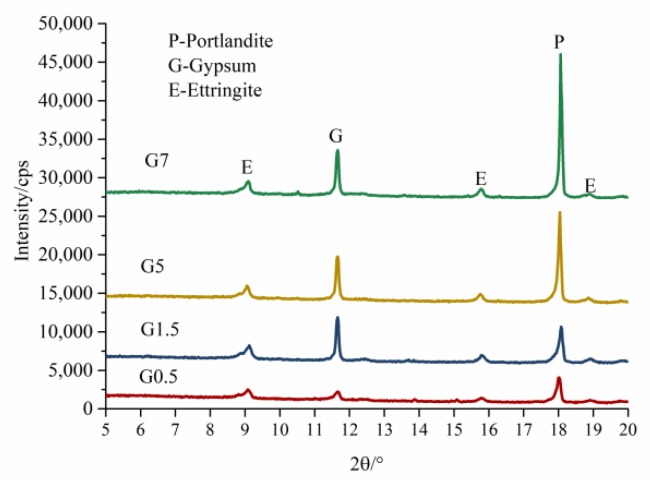
XRD patterns of paste in concrete with gypsum-contaminated coarse aggregate at 360 days.

To detect the existence of gypsum in mortar during the internal sulfate attack, while avoiding the effects of quartz and dolomite peaks in the XRD, the XRD results from 5° to 20° are shown in Figure 10 and Figure 11, for the most intensive peak of gypsum within 20° in the XRD patterns. 

Figure 10 shows a comparison of the X-ray diffractograms of concrete samples with different amounts of SO_3_ in sand at 56, 240, and 360 days. The peaks of ettringite (E), portlandite (P), and gypsum (G) are highlighted in the graph. The results indicate that the mortar in concrete had no gypsum when the SO_3_ content in the sand was less than 1.5% at 56 days. Note that there were apparently no peaks corresponding to gypsum in the FG3 specimen at 240 days. Moreover, for the FG5 and FG7 samples, there was also a certain amount of gypsum in mortar until 360 days. It was clear that during the process of ISA, the gypsum in concrete could be dissolved.

Figure 11 represents the X-ray patterns of concrete containing SO_3_ in coarse aggregate at 360 days. It is worth mentioning that all concrete with gypsum coarse aggregate incorporated had gypsum for 360 days during the internal sulfate attack. This provides evidence that the dissolution rate of gypsum in gypsum-contaminated aggregate with bigger particle size is relatively slower.

**Figure 12:**
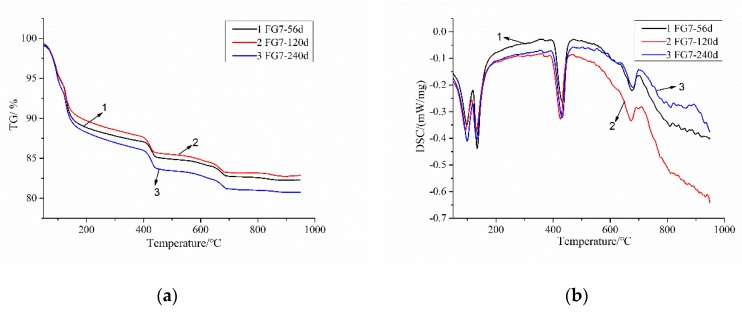
Thermogravimetry–differential scanning calorimetry (TG–DSC) curves of the FG7 specimen at the ages of 56, 120, and 240 days. (**a**) TG; (**b**) DSC.

The XRD results show that, during ISA the gypsum dissolved when the SO_3_ content was less than 3% in sand. For the higher contents of SO_3_ in FG5 and FG7, the new gypsum phase would be formed by the dissolution of SO_3_. To solve these problems, the TG–DSC analysis of FG7 specimen was researched. Figure 12 shows the TG–DSC curves of the FG7 samples at ages of 56, 120, and 240 days. As shown in Figure 12b, the temperatures of 80–120, 120–150, and 390–470 °C correspond to the decomposition of AFt (3CaO·Al_2_O_3_·3CaSO_4_·32H_2_O), gypsum (CaSO_4_·2H_2_O), and portlandite (Ca(OH)_2_), respectively. During heating from 80 to 120 °C, 26 mol of water will be removed from AFt [18]. The amount of AFt in the specimen can be calculated by Equation (2). According to the mass loss (at 120–150 °C and 390–470 °C) caused by the decomposition of CaSO_4_·2H_2_O and Ca(OH)_2_ (shown in Figure 12a), the quantities of gypsum and portlandite can be calculated using Equation (3) and Equation (4).

(2) $$AFt\left(\%\right)=W_{AFt}\cdot \frac{M_{AFt}}{26M_{H}}$$

(3) $$G\left(\%\right)=W_{G}\cdot \frac{M_{G}}{2M_{H}}$$

(4) $$P\left(\%\right)=W_{P}\cdot \frac{M_{P}}{2M_{H}}$$

where W_AFt_, W_G_, and W_P_ correspond to the mass loss in percentage attributable to AFt, gypsum, and portlandite dehydration, respectively, and M_AFt_, M_G_, and M_P_ are the molecular weights of AFt, gypsum, and portlandite, respectively. M_H_ is the molecular weight of H_2_O. 

**Figure 13:**
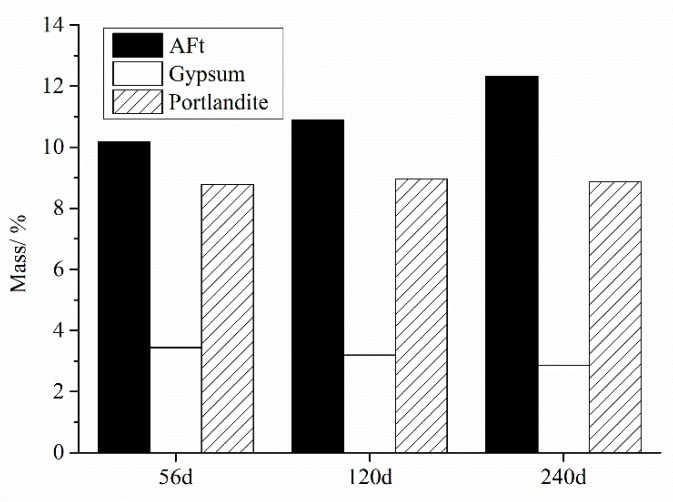
Estimated quantities of ettringite (Aft) and gypsum at 56, 120, and 240 days in concrete.

Figure 13 shows the estimated quantities of AFt and gypsum at 56, 120, and 240 days. The results show that the amount of gypsum phase decreased and the quantity of AFt increased during the ISA. Additionally, the amount of portlandite stayed relative stable throughout the whole process.

### 5.5. WP Dissolution in Concrete

**Figure 14:**
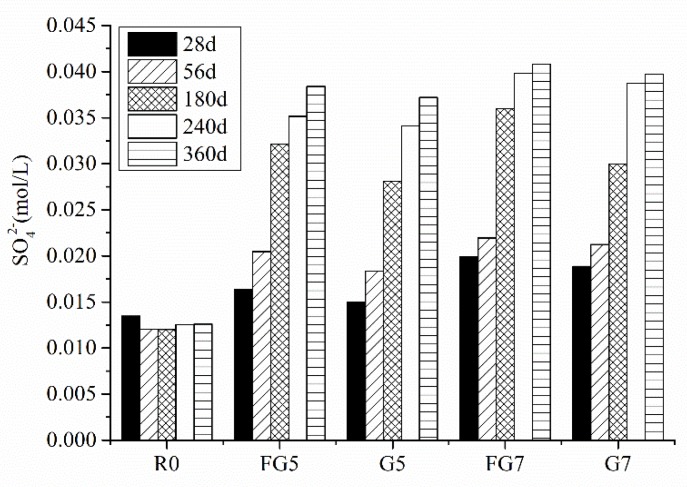
Concentration of SO_4_^2−^ in the pore solution at different ages.

Figure 14 shows the concentration of SO_4_^2−^ in the pore solution in concrete at different ages. As shown in Figure 14, concrete with 5% and 7% SO_3_ in aggregate showed a higher concentration of sulfate ions as compared to the reference sample at all ages. The sulfate concentration increased with an increase in the gypsum content in specimens. Figure 14 also indicates that the SO_4_^2−^ dissolved more easily for aggregate with a smaller particle size, which is consistent with the results for the expansion and compressive strength of samples. Furthermore, for the FG7 specimen, the concentration of SO_4_^2−^ was 0.0408 mol/L, which exceeded the minimum concentration of sulfate ions (1400 mg/L) to form gypsum in solution [11]. However, this number is much smaller than 8000 mg/L, the sulfate ion concentration required for external sulfate attack to form gypsum [12]. Additionally, as the mineral analysis results showed that portlandite did not decrease, this means that there was no new phase of gypsum formed. 

### 5.6. Microstructure

**Figure 15:**
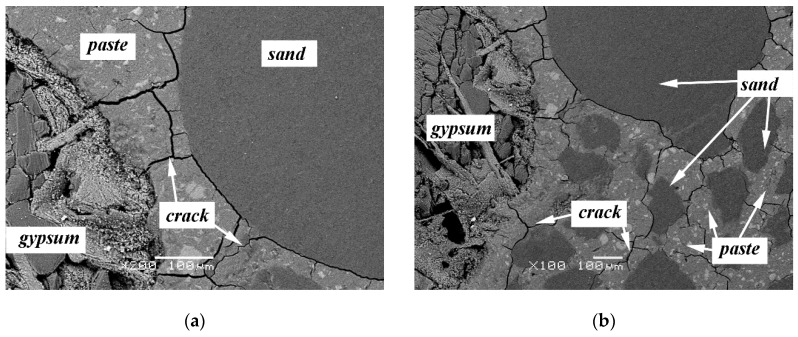
SEM photographs of cracking behavior of concrete with 5% SO_3_ in sand: (**a**) 120 days; (**b**) 240 days.

**Figure 16:**
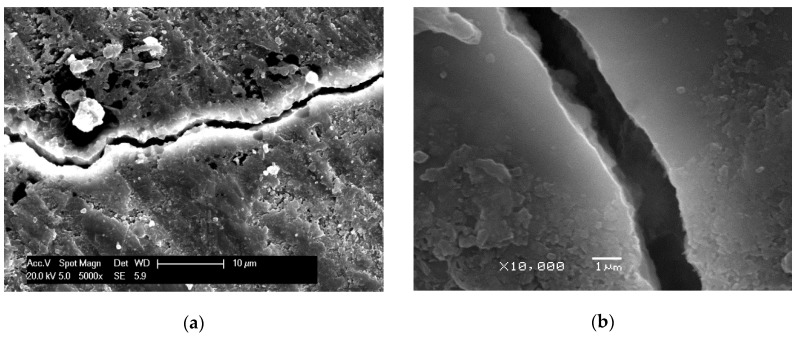
SEM photographs of crack width in concrete: (**a**) 120 days; (**b**)240 days.

**Figure 17:**
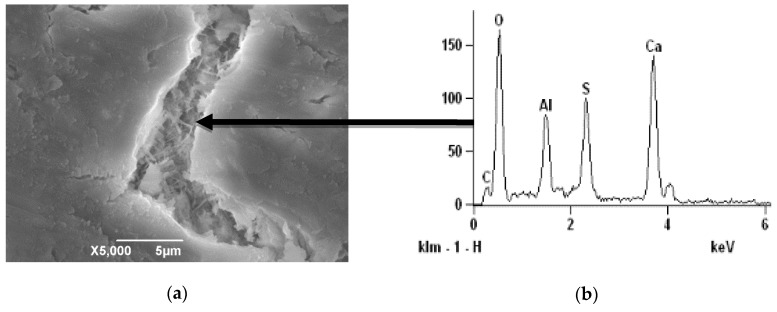
SEM images of cracking and EDS image of product in crack. (**a**) SEM; (**b**) EDS.

SEM was applied to observe the microstructure of mortar with 5% SO_3_ in sand. Figure 15 shows the cracking behavior of concrete with 5% SO_3_ in sand at 120 and 240 days. It is shown that with the development of ISA, the fracture firstly extended from the gypsum to the paste. Then, the crack extended around the sand, and finally, the sand separated from the paste completely. Figure 16 shows the crack width change during the ISA. The picture shows that the crack width increased significantly. Additionally, in the crack, the needle ettringite crystals oriented their growth in the surface between the paste and aggregate, as shown in Figure 17. Thus, the formation of ettringite was the cause of ISA in the concrete with gypsum-contaminated aggregate.

## 6. Conclusions

The deterioration process of concrete exposed to internal sulfate attack was investigated. The main conclusions are as follows:

- With an increase in the SO_3_ content in aggregate, the concrete expansion increased, and the compressive strength decreased. However, if the SO_3_ content was lower than 1.5% and 3% in fine aggregate and coarse aggregate, respectively. Although, the expansion was not obvious, and the compressive strength of samples was above the initial strength of the reference specimen. The critical values of SO_3_ content in fine and coarse aggregate were found to be 1.25% and 3%, respectively. At values above these harmful expansion and large strength loss appear.
- SO_4_^2−^ dissolved more easily when the particle size of aggregate was smaller. When the SO_3_ content in the sand was less than 1.5% at 56 days, all the gypsum was dissolved. That was the reason why the expansion of specimens with a smaller SO_3_ content (1.25% for fine aggregate and 3% for coarse aggregate) was not significant.
- The concentration of sulfate ions in the pore solution of concrete was not enough to form a new phase of gypsum when the SO_3_ content was less than 7% in aggregate. For the internal sulfate attack, the main reaction product is ettringite, which causes the expansion of specimens.
- The fracture mode of internal sulfate attack was the crack extending from gypsum particles to paste, which made the aggregate separate from the paste.
- In this paper, only TG–DSC was used to analyze the content of major phases, and no other methods were combined. This paper only studied the changes in compressive strength and expansion performance of concrete containing gypsum aggregates for 360 days. To study whether it is safe to use gypsum aggregates in concrete, other durability tests are needed. In addition, there has been lack of evaluation on methods that can be used to determine the safe dosage of gypsum aggregate in concrete at home and abroad, which will be the focus of our follow-up work.

## Figures and Tables

**Table 1:** Chemical compositions of P·II 52.5 Portland cement.

SiO_2_	CaO	Al_2_O_3_	Fe_2_O_3_	MgO	Na_2_O	SO_3_
19.46	63.23	4.53	2.80	2.41	0.13	3.31

**Table 2:** Chemical compositions of gypsum-contaminated aggregate.

SiO_2_	CaO	Al_2_O_3_	Fe_2_O_3_	MgO	K_2_O	Na_2_O	SO_3_	LOI
15.83	17.21	6.71	4.64	11.59	2.87	0.26	18.03	21.08

**Table 3:** Chemical compositions of the white part (WP) in aggregate.

SiO_2_	CaO	Al_2_O_3_	MgO	Fe_2_O_3_	SO_3_	K_2_O	Na_2_O	TiO_2_	LOI
0.19	35.43	0.08	4.05	0.02	51.36	0.01	0.01	0.01	12.79

**Table 4:** Mix design of concretes (kg/m^3^).

No.	SO_3_ by Weight of Aggregate/%	Cement	Water	Fine Aggregate		Coarse Aggregate	
				River Sand	Gypsum Sand	Limestone	Gypsum Stone
R0	0	420	189	735	0	1110	0
G0.5	0.5	420	189	735	0	1058	52
G1	1					1006	104
G1.5	1.5					954	156
G3	3					798	312
G5	5					590	520
G7	7					382	728
FG0.5	0.5	420	189	683	52	1110	0
FG1	1			631	104		
FG1.5	1.5			579	156		
FG3	3			423	312		
FG5	5			215	520		
FG7	7			7	728		
